# Intranasal HD-Ad vaccine protects the upper and lower respiratory tracts of hACE2 mice against SARS-CoV-2

**DOI:** 10.1186/s13578-021-00723-0

**Published:** 2021-12-08

**Authors:** Huibi Cao, Juntao Mai, Zhichang Zhou, Zhijie Li, Rongqi Duan, Jacqueline Watt, Ziyan Chen, Ranmal Avinash Bandara, Ming Li, Sang Kyun Ahn, Betty Poon, Natasha Christie-Holmes, Scott D. Gray-Owen, Arinjay Banerjee, Karen Mossman, Rob Kozak, Samira Mubareka, James M. Rini, Jim Hu, Jun Liu

**Affiliations:** 1 Translational Medicine Program, Hospital for Sick Children Research Institute, Toronto, ON Canada; 2 Department of Molecular Genetics, Faculty of Medicine, University of Toronto, Toronto, ON Canada; 3 Department of Laboratory Medicine and Pathobiology, Faculty of Medicine, University of Toronto, Toronto, ON Canada; 4 Combined Containment Level 3 Unit, Faculty of Medicine, University of Toronto, Toronto, ON Canada; 5 Vaccine and Infectious Disease Organization, University of Saskatchewan, Saskatoon, SK Canada; 6 Department of Veterinary Microbiology, University of Saskatchewan, Saskatoon, SK Canada; 7 Department of Biology, University of Waterloo, Waterloo, ON Canada; 8 Department of Medicine Institute for Infectious Disease Research, McMaster Immunology Research Center, McMaster University, Hamilton, ON Canada; 9 Sunnybrook Heath Sciences Centre, Toronto, ON Canada; 10 Department of Biochemistry, Faculty of Medicine, University of Toronto, Toronto, ON Canada

**Keywords:** COVID-19, HD-Ad, Nasal delivery, SARS-CoV2, Vaccine

## Abstract

**Background:**

The ongoing COVID-19 pandemic has resulted in 185 million recorded cases and over 4 million deaths worldwide. Several COVID-19 vaccines have been approved for emergency use in humans and are being used in many countries. However, all the approved vaccines are administered by intramuscular injection and this may not prevent upper airway infection or viral transmission.

**Results:**

Here, we describe a novel, intranasally delivered COVID-19 vaccine based on a helper-dependent adenoviral (HD-Ad) vector. The vaccine (HD-Ad_RBD) produces a soluble secreted form of the receptor binding domain (RBD) of the SARS-CoV-2 spike protein and we show it induced robust mucosal and systemic immunity. Moreover, intranasal immunization of K18-hACE2 mice with HD-Ad_RBD using a prime-boost regimen, resulted in complete protection of the upper respiratory tract against SARS-CoV-2 infection.

**Conclusion:**

Our approaches provide a powerful platform for constructing highly effective vaccines targeting SARS-CoV-2 and its emerging variants.

**Supplementary Information:**

The online version contains supplementary material available at 10.1186/s13578-021-00723-0.

## Introduction

The ongoing COVID-19 pandemic, caused by severe acute respiratory syndrome coronavirus 2 (SARS-CoV-2) [1], has resulted in more than 180 million confirmed cases and at least 4 million deaths worldwide. The development of safe and effective vaccines against SARS-CoV-2 is a global health priority and a number of vaccine platforms have already been tested [2]. These include inactivated virus [3, 4], naked DNA delivered by electroporation [5], mRNA delivered by lipid nanoparticles [6–9], viral vectors such as nonreplicating adenovirus [10–17], replication-competent vesicular stomatitis virus (VSV) [18] or yellow fever virus [19], and recombinant protein delivered by nanoparticles [20, 21]. To date, a number of vaccines have been evaluated in phase III clinical trials including the mRNA vaccines from Pfizer-BioNTech [22] and Moderna [23], the ChAd vaccine from AstraZeneca [24], and the rAd26 and rAd5 vaccines produced and tested in Russia [25].

Unlike the vectors used in the currently approved Ad-based SARS-CoV-2 vaccines [24–26], helper-dependent adenoviral vectors (HD-Ad) are third generation vectors completely devoid of adenoviral coding sequences [27]. They were designed to eliminate the expression of unwanted adenoviral proteins [28, 29] and they have been used primarily in preclinical tests of in vivo gene delivery for the treatment of inherited genetic diseases [28, 30]. HD-Ad has also been used in clinical trials of gene therapy [31]. Since HD-Ad does not integrate into the host genome, it also eliminates the risk of introducing chromosomal mutations. The absence of adenoviral protein expression minimizes the host inflammatory and immune responses to the vector thereby reducing toxicity and allowing for longer-term transgene expression in host tissues or organs [32–36]. In addition, HD-Ad has a high cloning capacity (up to 36 kb) for transgenes, making it possible to deliver large genes or multiple genes in one vector. These features, in conjunction with its excellent safety profile, make HD-Ad an attractive platform for the development of a SARS-CoV-2 vaccine.

All of the COVID-19 vaccines approved, to date, are delivered by intramuscular injection [22–26], an administration route that may not protect the upper respiratory tract and stop viral shredding and transmission [37]. In contrast, intranasal administration elicits a local immune response, including secretory IgA antibodies, that can provide protection at or near the site of infection of respiratory pathogens [38]. Here, we describe a novel, nasally administered COVID-19 vaccine based on an HD-Ad vector. The vaccine, HD-Ad_RBD, produces a soluble, secreted form of the receptor binding domain (RBD) of the SARS-CoV-2 S-protein. The RBD mediates virus binding to the host receptor, human angiotensin converting enzyme 2 (hACE2), and antibodies against it represent the most common route to host-mediated viral neutralizing [39–42]. Using K18-hACE2 mice [43], we find that intranasal delivery of HD-Ad_RBD elicits robust mucosal and systemic immunity, as well as complete protection of the upper airway in SARS-CoV-2 viral challenge experiments.

## Results

### Cloning and expression of a secreted form of the SARS-CoV-2 RBD

**Fig. 1:**
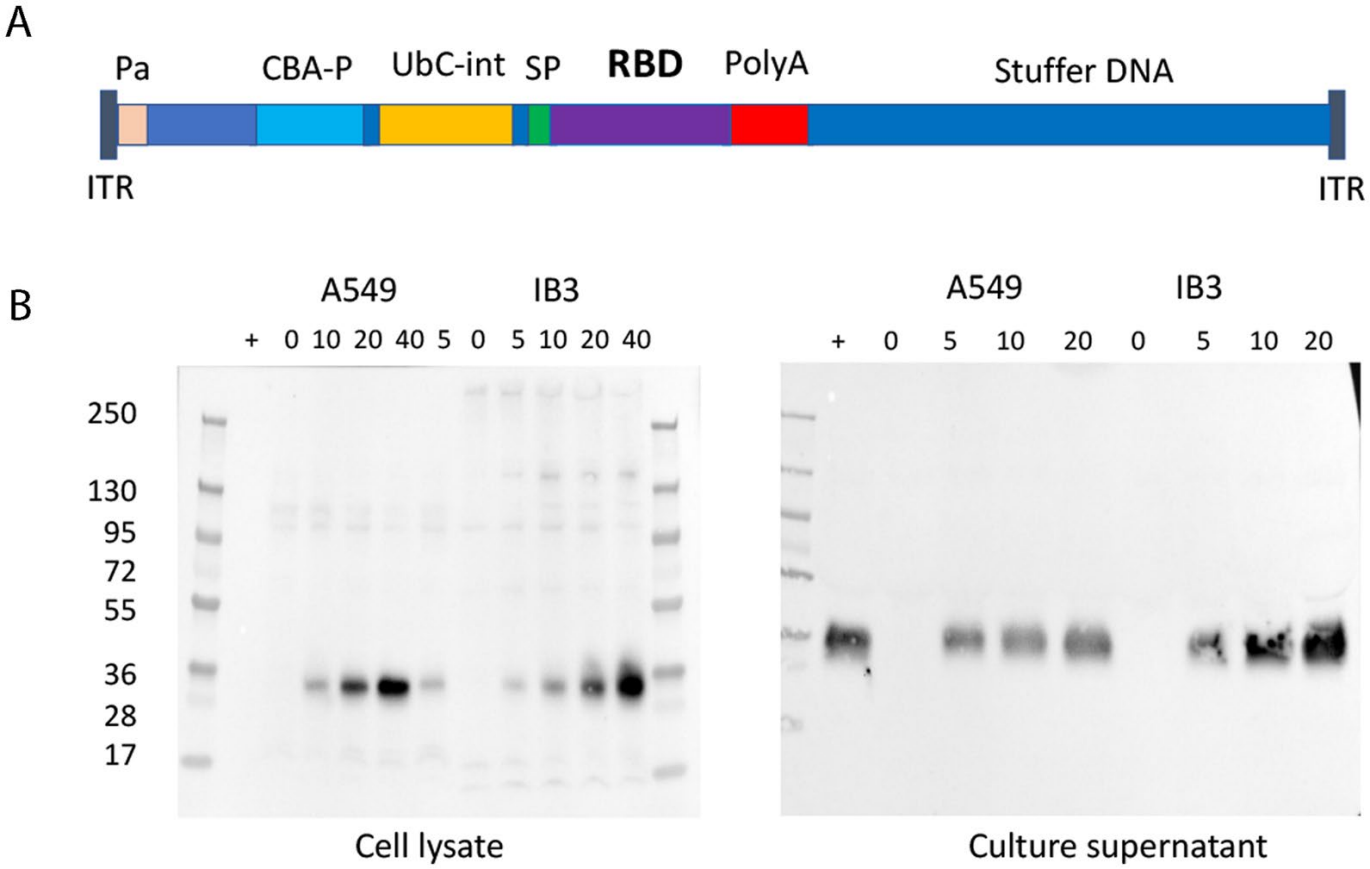
Construction of the HD-Ad_RBD vaccine. **A** HD-Ad_RBD vaccine schematic. ITR, adenovirus inverted terminal repeat; Pa, adenovirus packaging signal; CBA-P, chicken beta actin gene promoter with CVM enhancer; UbC-int, the first intron of the human ubiquitin C gene; SP, the human cystatin-S signal peptide; RBD, receptor binding domain of the SARS-CoV-2 spike protein; PolyA, transcription termination signal of the Bovine Growth Hormone gene; Stuffer DNA, noncoding human DNA used to make the vector genome large enough to be packaged. **B** Western blot analysis of RBD expression and secretion. Epithelial cells A549 and IB3 were transfected with HD-Ad_RBD at the indicated dosages and cell lysates and culture supernatants were prepared and subjected to Western blot analysis using anti-RBD antibodies

The RBD of the SARS-CoV-2 S-protein was codon optimized (Additional file 2: Table S1) and expressed from the chicken beta-actin (CBA) promoter with a cytomegalovirus (CMV) enhancer, to increase transcription, and the first intron of the human *UbC* gene to increase mRNA stability (Fig. 1A). A DNA sequence encoding the 20-amino acid signal peptide of the human cystatin S protein was included upstream of the coding sequence of the RBD, which allows for RBD secretion. The BGH poly A tail was used to terminate transcription.

To examine the expression and secretion of the RBD, epithelial cells (A549 and IB3) were transfected with HD-Ad_RBD at different dosages and the cell lysates and culture supernatants were analyzed by Western blot. The results showed that the RBD was expressed at high levels in both cell lines in a dose-dependent manner and that approximately 90% of the RBD was found in the culture supernatant (Fig. 1B). The secreted transgene product delivered by HD-Ad vectors is known to reach both airway fluid and the blood circulation system [30] and, as such, the secreted RBD is expected to reach antigen presenting cells, locally and systemically, to induce antigen-specific immune responses.

### HD-Ad_RBD induces robust mucosal and systemic immunity

**Fig. 2:**
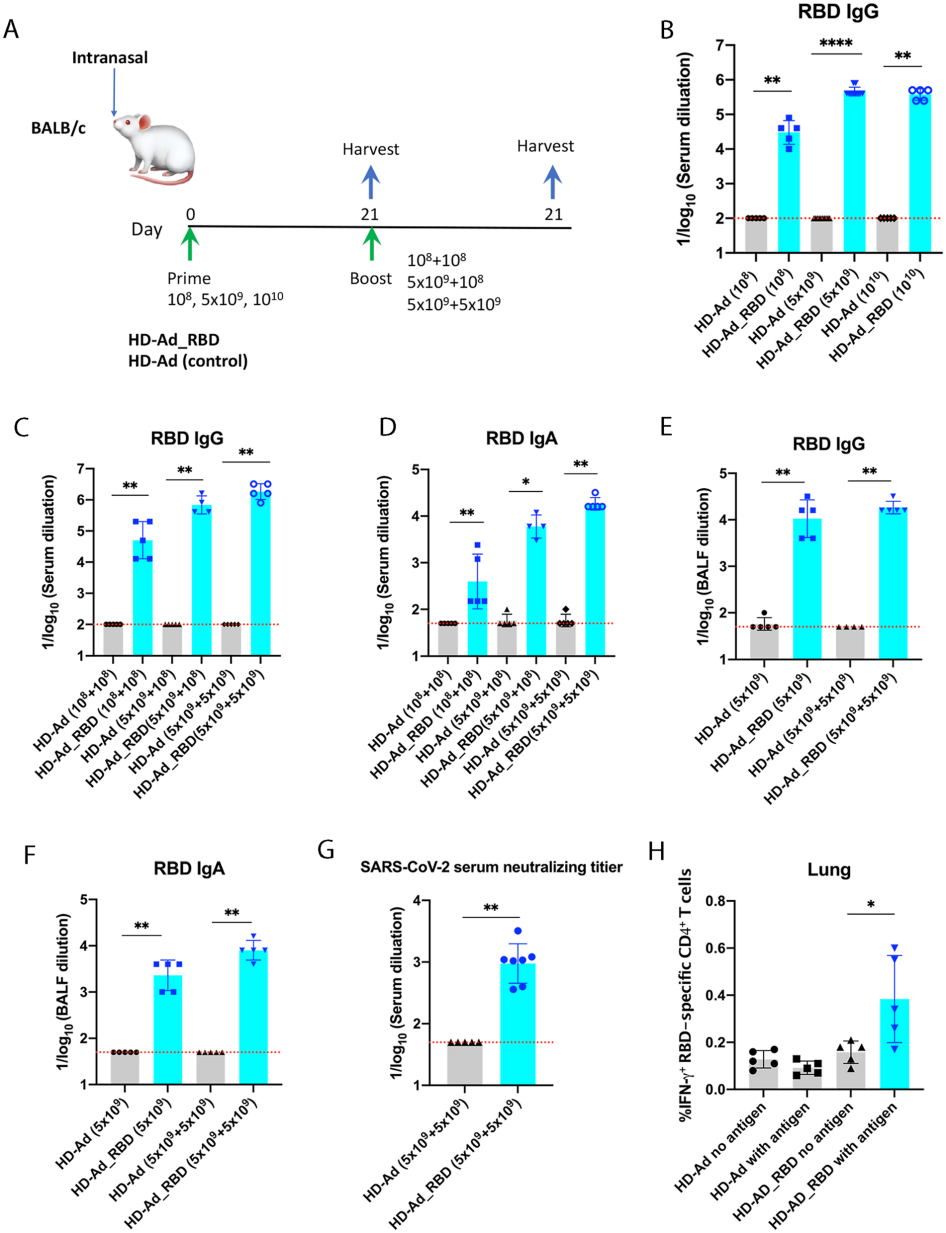
HD-Ad_RBD induces high levels of IgG, IgA and neutralizing antibody. **A** Experimental scheme. BALB/c mice were immunized with HD-Ad_RBD or HD-Ad via an intranasal route. **B** Antibody responses after single vaccination with different doses. RBD-specific IgG in sera were measured by ELISA. **C**, **D** Antibody responses after prime-boost vaccination with different doses. RBD-specific IgG (**C**) and IgA (**D**) in sera were measured by ELISA. **E**, **F** Antibody responses detected in BALs. BALs from single (5 × 10^9^) or prime-boost vaccinated (5 × 10^9^ + 5 × 10^9^) mice were collected and RBD-specific IgG (**E**) and IgA (**F**) were measured by ELISA. **G** SARS-CoV-2 neutralizing activity of sera. **H** Intracellular cytokine (IFN-γ) staining and flow cytometry analysis of CD4^+^ T cells. Harvested lung cells were stimulated with or without purified RBD (10 μg/ml) for 12 h at 37 °C and subjected to ICS analysis. In all figures, each dot represents an animal. Bars and errors represent the geometric mean with geometric SD. The red dotted lines indicate the limit of detection (LOD) of the assays. Statistical analyses were performed by Mann–Whitney test: *p < 0.05; **p < 0.01; ***p < 0.001

To examine the immune responses induced by HD-Ad_RBD, we intranasally immunized BALB/c mice (*n* = 5) with three different doses of HD-Ad_RBD. Three weeks later these mice were sacrificed and sera collected (Fig. 2A). ELISA analysis of the sera showed high levels of RBD-specific IgG in all three groups of mice immunized with HD-Ad_RBD, whereas low, if any, RBD-specific IgG were detected in mice immunized with the HD-Ad vector control, which lacks a transgene (Fig. 2B). Animals vaccinated with 10^8^, 5 × 10^9^ and 10^10^ HD-Ad_RBD viral particles had reciprocal geometric mean titers (GMT) of 30,314, 459,479, and 378,929, respectively. This result indicates a dose-dependent response and that 5 × 10^9^ viral particles is the optimal dose for HD-Ad_RBD vaccination.

We next tested a prime-boost intranasal vaccination regimen. Based on the results of the single dose vaccinations, we chose 10^8^ and 5 × 10^9^ HD-Ad_RBD viral particles for the prime immunizations, followed 3 weeks later by boost vaccinations at the same dose (Fig. 2A). A lower boost dose (10^8^ viral particles) was also tested in the 5 × 10^9^-primed group. Three weeks after the second vaccination, the animals were sacrificed and sera and bronchoalveolar lavages fluids (BALFs) were collected.

Boosting with 10^8^ HD-Ad_RBD viral particles increased the IgG titer ~ 1.5-fold relative to the single 10^8^ vaccination, and the IgG reciprocal GMTs for the 10^8^ prime-boost group, and the 5 × 10^9^-prime/10^8^-boost group were 50,476 and 688,862, respectively (Fig. 2C). Remarkably, the IgG reciprocal GMT in the 5 × 10^9^ prime-boost group increased fourfold compared to that of the single vaccination and reached 1,837,920 (Fig. 2C).

High levels of RBD specific IgA were also detected in the sera of the boosted animals, with the 5 × 10^9^ prime-boost group reaching a reciprocal GMT of 18,379 (Fig. 2D).

We also detected high RBD-specific IgG and IgA levels in BALFs. For the 5 × 10^9^ prime-boost group, the IgG and IgA reciprocal GMTs were 18,379 and 8,000, respectively (Fig. 2E, F).

Similarly, there was a dose-dependent increase in the neutralizing activity of the sera against the SARS-CoV-2 virus (Fig. 2G). The reciprocal 50% inhibition dilution (ID_50_) GMTs of the neutralizing antibody in the 5 × 10^9^-prime/10^8^-boost group and the 5 × 10^9^ prime-boost group were 378 and 948, respectively. Neutralizing activity was not detected in BALFs.

Finally, we also detected an increase in IFN-γ producing CD4^+^ T cells in the lungs of animals vaccinated with 5 × 10^9^ HD-Ad_RBD viral particles (prime and boost) compared to the control groups, indicating that the Th1 response was activated (Fig. 2H).

### HD-Ad_RBD protects hACE2 mice against SARS-CoV-2 infection

**Fig. 3:**
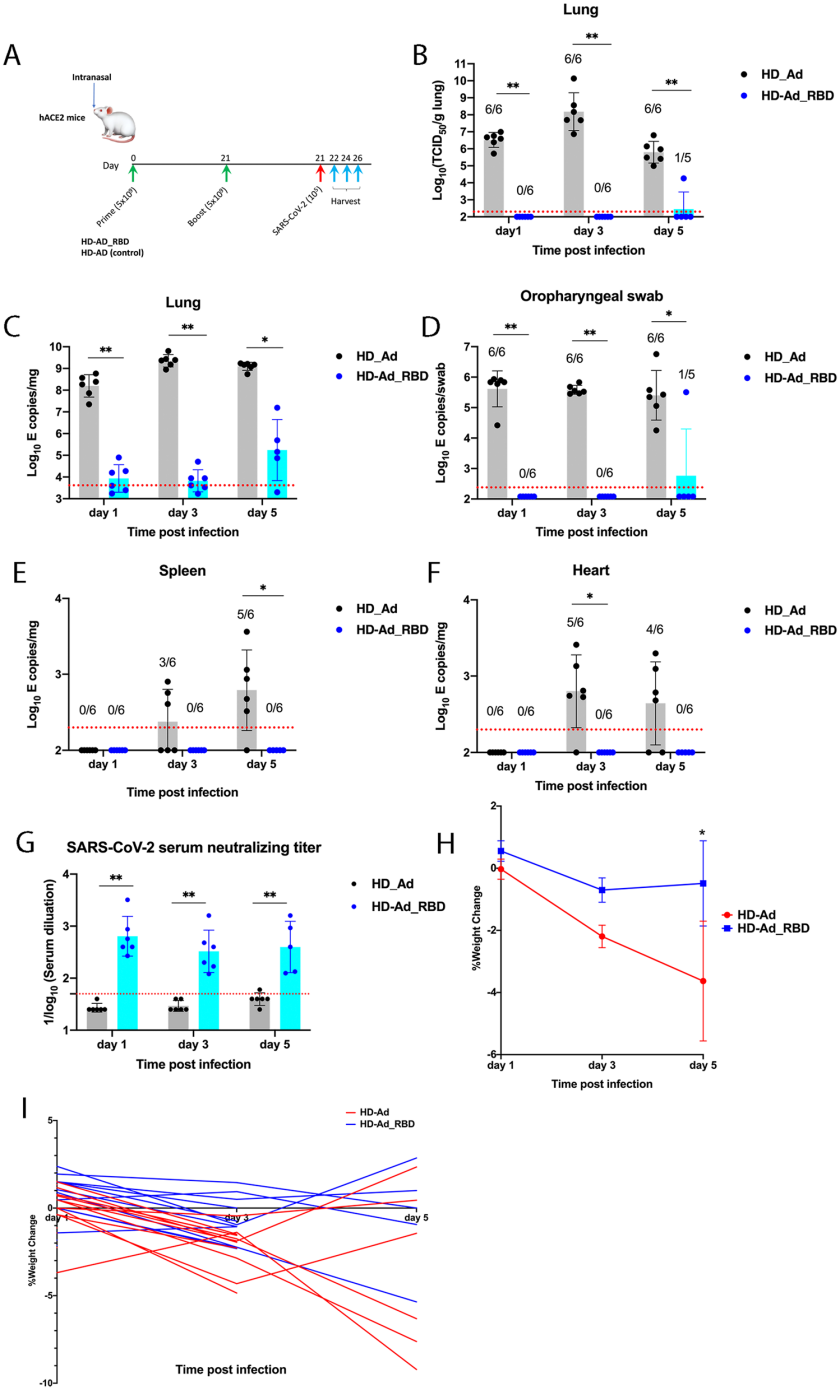
HD-Ad_RBD protects the upper and lower respiratory tracts against SARS-CoV-2 infection. **A** Experimental scheme. hACE2 mice were immunized intranasally with HD-Ad_RBD using a prime-boost regimen at a dose of 5 × 10^9^ viral particles. Three weeks after the second vaccination, the animals were challenged intranasally with 10^5^ TCID_50_ of SARS-CoV-2. At day 1, 3 and 5 post-infection, mice were euthanized, and the lungs, spleen and heart were harvested for viral burden and cytokine analysis. **B** Titers of infectious SARS-CoV-2. The number of infectious virus particles in the lungs were determined by CPE assays. **C**–**F** RNA levels of SARS-CoV-2 determined by qRT-PCR. Viral RNA levels in the lung, spleen, heart, and oropharyngeal swabs were measured at the indicated time points by qRT-PCR. **G** Neutralizing activity of sera against SARS-CoV-2. Sera from mice at different time points after SARS-CoV-2 infection were collected and the levels of neutralizing antibody were measured. Each dot represents an animal. Bars and errors represent the geometric mean with geometric SD. The red dotted lines indicate the limit of detection (LOD) of the assays. Statistical analyses were performed by Mann–Whitney test: *p < 0.05; **p < 0.01; ***p < 0.001. **H** The percentage of weight loss (mean ± SEM) of animals at the indicated time points after SARS-CoV-2 infection. Statistical analysis was performed by two-way ANOVA: *p < 0.05. **I** The percentage of weight loss of individual animal at the indicated time points post infection

To examine the protective efficacy of HD-Ad_RBD, we intranasally immunized K18-hACE2 transgenic mice with HD-Ad_RBD (*n* = 17) or the HD-Ad vector alone (sham control, *n* = 18). The K18-hACE2 transgenic mouse model was generated by McCray et al. [44] by expressing human ACE2, the receptor for SARS-CoV-2, using the K18 gene expression cassette developed by one of our laboratories [45]. The hACE2 mice received a prime immunization of 5 × 10^9^ viral particles of HD-Ad_RBD or HD-Ad at day 1. At day 21, the mice received a boost immunization, of the vaccine or the control, at the same dose. At day 21 after the second immunization, the mice were intranasally challenged with SARS-CoV-2 at 10^5^ 50% tissue culture infectious dosage (TCID_50_). At day 1, 3 and 5 post infection, mice were euthanized and lungs, spleen and heart were harvested for viral burden and cytokine analysis (Fig. 3A).

Notably, there was no detectable infectious virus in the lungs of 16 of the mice (*n* = 17) immunized with HD-Ad_RBD as determined by the TCID_50_ assay, whereas high levels of the infectious virus were detected in all the mice (*n* = 18) vaccinated with the vector control (Fig. 3B). One mouse immunized with HD-Ad_RBD showed low but detectable levels of infectious virus and we suggest that it may not have been properly vaccinated as the mice sometimes sneezed out the vaccine solution during intranasal inoculation. Using SARS-CoV-2 *E* gene specific primers, we detected very high levels of viral RNA (10^8^ to 10^9^ copies/mg) in the lungs of mice vaccinated with the HD-Ad vector control. In contrast, the viral RNA levels were reduced by > 4 log_10_ in 16 out of the 17 mice vaccinated with HD-Ad_RBD (Fig. 3C). The very low RNA levels in the lungs of the mice vaccinated with HD-Ad_RBD may reflect an inability of the SARS-CoV-2 challenge dose to replicate in these animals. This suggestion is consistent with the following two lines of reasoning. First, there was a significant increase (> 1 log_10_) in both the infectious viral titer and the viral RNA copy number in the lungs of the control mice at day 3 compared to day 1, an indication of substantial viral replication in these mice (Fig. 3B, C). In contrast, no infectious virus was detected in the HD-Ad_RBD vaccinated mice and the viral RNA level remained constant at day 1 and 3 post-infection (Fig. 3B, C). Second, the low SARS-CoV-2 RNA levels (~ 10^4^ copies/mg) observed at day 1 and 3 in the vaccinated animals are similar to that observed at these time points in SARS-CoV-2 infection experiments in BALB/c and C57BL/6 mice (which lack hACE2) where replication of the input virus is not possible [46].

Remarkably, intranasal delivery of HD-Ad_RBD provided effective protection of the upper respiratory tract as judged by the absence of measurable viral RNA in the oropharyngeal swabs (Fig. 3D). Only the same abovementioned mouse that showed infection in the lungs showed a detectable level of viral RNA. All mice in the control group exhibited high levels of viral RNA (10^6^ copies/swab). We also detected no measurable or very low levels of viral RNA in the heart and spleen of the mice vaccinated with HD-Ad_RBD (Fig. 3E, F).

The protection against SARS-CoV-2 in HD-Ad_RBD vaccinated animals is likely due to high neutralizing antibody levels present at the time of challenge. To test this we collected, shortly after SARS-CoV-2 challenge (day 1, 3 and 5 post-infection), sera from both HD-Ad_RBD vaccinated animals and sham (HD-Ad) vaccinated animals. In the animals vaccinated with HD-Ad_RBD, high levels of neutralizing antibody against SARS-CoV-2 were detected, with reciprocal ID_50_ GMTs ranging from 328 to 640 (Fig. 3G); these values are in the same range as the neutralizing antibodies detected in the vaccinated but not challenged animals (Fig. 2G). In animals vaccinated with HD-Ad, only low or undetectable levels of neutralizing antibodies were found after SARS-CoV-2 challenge (Fig. 3G). Taken together, these observations are consistent with the idea that the protection observed in the HD-Ad_RBD vaccinated animals is due to existing high levels of neutralizing antibodies, not antibodies elicited by the SARS-CoV-2 challenge itself.

For sham vaccinated animals, there was on average 4% of weight loss at day 5 post infection (Fig. 3H, I). The modest level of weight loss is consistent with the relative short period (5 days) of infection experiments performed. Importantly, in majority of animals vaccinated with HD-Ad, the weight loss was negligible (< 1%), and at day 5 post infection, there was a significant difference on the average weight loss between the vaccinated group and control group (Fig. 3H, I).

### HD-Ad_RBD vaccination protects mice against SARS-CoV-2-induced inflammation in the lung

We next examined the effect of the HD-Ad_RBD on SARS-CoV-2-induced lung inflammation. Lung tissue at day 3 post infection was chosen for this analysis. mRNA levels of several proinflammatory cytokines and chemokines were measured and normalized against samples prepared from naïve mice, mice that were vaccinated with the HD-Ad vector control but not challenged with SARS-CoV-2.

**Fig. 4:**
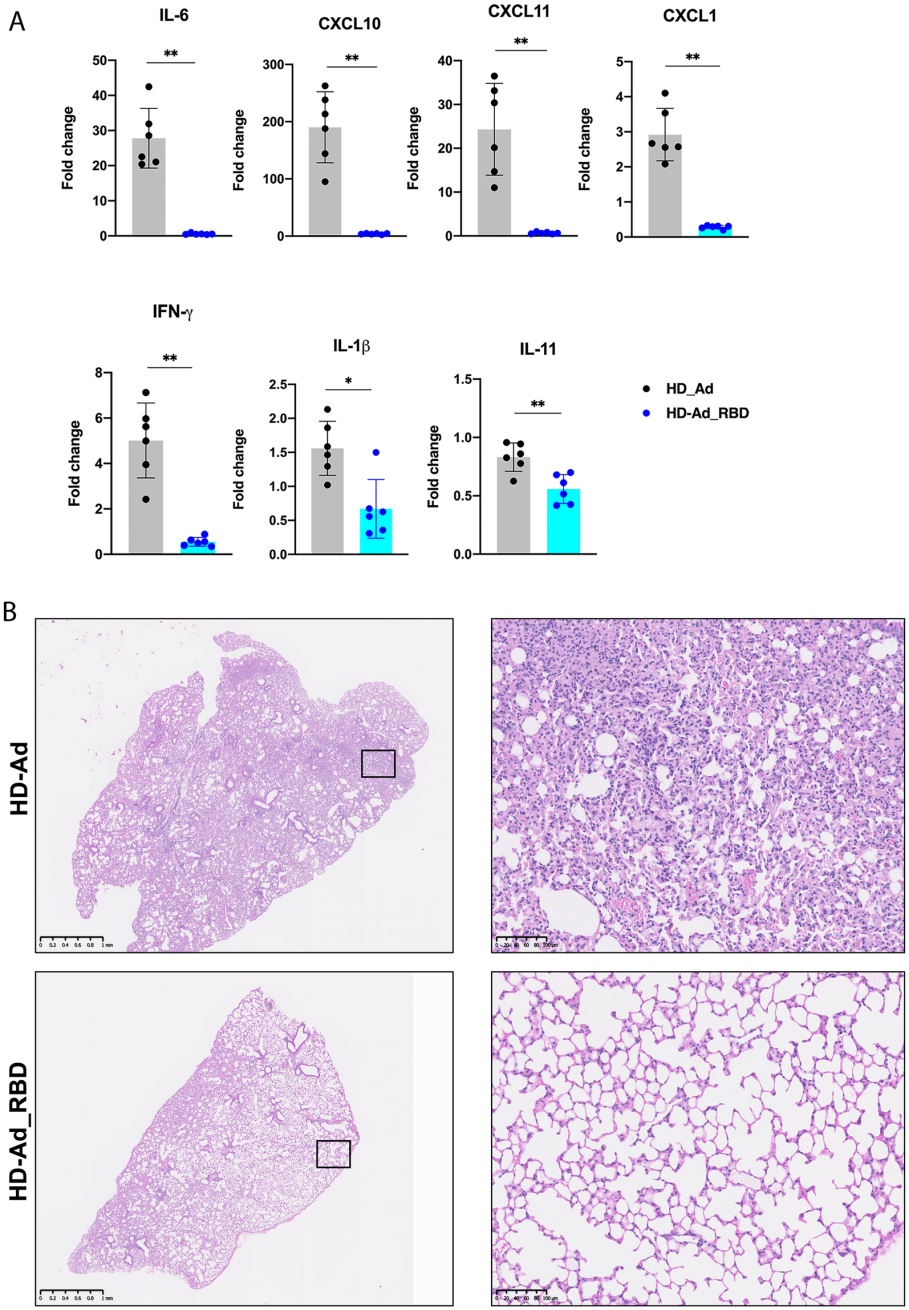
HD-AD_RBD prevents lung inflammation after viral challenge. **A** Fold change in gene expression of the indicated cytokines and chemokines from lung homogenates at day 3 post infection was determined by qRT-PCR after normalization to *GAPDH* levels and comparison with the naive unchallenged control. Each dot represents an animal. Bars and errors represent the mean with SD. Statistical analyses were performed by Mann–Whitney test: *p < 0.05; **p < 0.01; ***p < 0.001. **B** H&E staining of lung sections of hACE2 mice at day 3 post infection. Images show low- (left, scale bar, 1 mm) and high- (right, scale bar, 100 µm) power magnification. Images are representative of *n* = 6 per group

In the mice that were vaccinated with the HD-Ad vector control and challenged by SARS-CoV-2, there was a dramatic increase in the mRNA levels of *IL-6*, *CXCL10*, and *CXCL11* relative to that observed in the naïve mice (Fig. 4A). However, in mice that were vaccinated with HD-Ad_RBD and challenged by SARS-CoV-2, these proinflammatory cytokines and chemokines remained at the same levels found in the naïve mice. In animals challenged by SARS-CoV-2, the mRNA levels of *CXCL1*, *IFN-γ*, *IL-1β* and *IL-11* were also significantly lower in the lung tissues of animals immunized with HD-Ad_RBD compared to the HD-Ad control (Fig. 4A).

We also performed histopathological analysis of the same lung tissues analyzed above (Fig. 4B). Consistently, the sham vaccinated mice challenged with SARS-CoV-2 showed massive infiltration of immune cells in alveolar space and interstitial location, which was largely absent in mice that were vaccinated with HD-Ad_RBD and challenged by SARS-CoV-2 (Fig. 4B).

Taken together, our results demonstrate that immunization with HD-Ad_RBD decreases both viral infection and consequent inflammation in the lungs of animals infected with SARS-CoV-2.

## Discussion

Reported here is a novel, nasally administered COVID-19 vaccine (HD-Ad_RBD) based on a helper-dependent adenoviral vector. It produces a soluble, secreted form of the SARS-CoV-2 RBD and tested in mice it elicited robust mucosal and systemic immune responses following a prime-boost regime (5 × 10^9^ viral particles, 3 week interval). Consistently high levels of antigen-specific IgA in both the BALFs (reciprocal GMT: 8000) and sera (reciprocal GMT: 18,379) of the vaccinated animals were observed, and high antigen-specific IgG (reciprocal GMT: 1,837,920) and neutralizing antibody (reciprocal ID_50_ GMT: 948) levels were observed in sera. Moreover, in intranasal SARS-CoV-2 challenge experiments (10^5^ TCID_50_), the vaccine prevented lung inflammation and provided complete protection of the upper airway as evidenced by the absence of virus or viral RNA at day 1 post-infection. Taken together, our data show that HD-Ad_RBD elicits a robust immune response and controls infection at the site of inoculation, outcomes that should prevent virus-induced disease and transmission.

These results can be compared with that of the currently approved COVID-19 vaccines, all of which are administered by intramuscular injection. The ChAdOx1 nCoV-19 vaccine, developed by the University of Oxford and AstraZeneca, is based on a replication-incompetent chimpanzee adenovirus and it was tested in rhesus macaques using a prime-boost regime (2.5 × 10^10^ viral particles, 4 week interval) administered intramuscularly [15]. Although the animals developed moderate neutralizing antibody titers (reciprocal ID_50_ GMT: 10–160) and were mostly protected from lung disease, viral replication in the upper respiratory tract was not controlled [15]. The mRNA-1273 vaccine developed by the National Institutes of Health (NIH) and Moderna was also tested in rhesus macaques using a prime-boost regimen (4 week interval) and intramuscular injection [7]. Doses of 10 μg and 100 μg were tested and at the higher dose, the vaccine induced high IgG (reciprocal GMT value of 36,386) and neutralizing antibody levels (reciprocal ID_50_ GMT: 1862). The animals were almost completely protected from SARS-CoV-2 infection in the lower respiratory tract (BALFs), but viral RNA was detected in the upper respiratory tract (nasal swabs) in three of the eight macaques on day 1 post-infection, and in one of the eight macaques on day 4 post-infection (at the 100 μg dose) [7]. A similar finding was observed when the mRNA-1273 vaccine was tested in mice [47]. The BNT162b2 mRNA vaccine developed by BioNTech and Pfizer was also tested in rhesus macaques at two doses (30 μg and 100 μg) using a prime-boost regimen and intramuscular injection [48]. High levels of neutralizing antibody (reciprocal ID_50_ GMT: 1689) were detected in the high-dose group and these animals were completely protected against SARS-CoV-2 infection in BALFs. However, high levels of viral RNA were detected in nasal swabs of five of the six vaccinated animals at day 1 post-infection, and in oropharyngeal swabs of three of the six animals at day 1 post-infection and two of the six animals at day 3 post-infection [48]. These studies show that although a robust immune response was elicited in all cases, none of the currently approved vaccines provided protection of the upper airway in viral challenge experiments. Our results with HD-Ad_RBD suggest that this outcome can be significantly improved by a nasally administered HD-Ad-based vaccine.

To our knowledge, there has been only one published study evaluating the intranasal delivery of a COVID-19 vaccine candidate. Hassan et al. compared intramuscular injection and intranasal delivery of a chimpanzee Ad-based (simian Ad-36) SARS-CoV-2 vaccine in mice [12]. They found that intranasal delivery induced the production of IgA and IgG in both sera and BALFs of the animals. In contrast, intramuscular injection induced the production of IgG in sera and at very low level in BALFs, but IgA was not detected in sera and BALFs, which resulted in less protection than intranasal delivery. In hACE2 mice, a single intranasal delivery of the vaccine (10^10^ viral particles) followed by challenge with SARS-CoV-2 resulted in very low levels of viral RNA in the nasal turbinates or washes, an observation suggesting almost complete protection of the upper respiratory tract against infection [12]. That study, combined with our findings, suggest that the production of IgA and IgG in airway (BALFs) likely contributes to protection of the upper respiratory tract. However, in the study by Hassan et al., SARS-CoV-2 at 10^3^ foci forming units (FFU) were used in the challenge experiment, a value lower than that (4 × 10^5^ FFU) used in their other experiments [12]. By comparison, our HD-Ad_RBD vaccine (prime-boost with 5 × 10^9^ viral particles, 3 week interval) resulted in protection of the upper airway after SARS-CoV-2 challenge with 10^5^ TCID_50_, a dose much higher than that of 10^3^ FFU.

In addition to the intranasal delivery route, the efficacy of our HD-Ad_RBD vaccine is also likely due to high antigen expression levels. Replication-deficient adenoviral vectors with one or more early genes deleted (the first- and second-generation Ad vectors) are among the most efficient vehicles for in vivo gene delivery and HD-Ad vectors are expected to perform even better. While the capsid proteins present in HD-Ad may still induce some levels of unwanted host immune responses, this effect is likely greatly reduced compared to the first- and second-generation Ad vectors since HD-Ad is devoid of adenoviral coding sequences. Supporting this, a phase I clinical trial using HD-Ad as a transgene delivery vehicle observed only a transient inflammatory response [31]. Therefore, the HD-Ad platform is expected to lead to longer-term transgene expression and reduced toxicity [49–51]. Western blot analysis showed that A549 and IB3 cells transfected with HD-Ad_RBD led to high levels of secreted RBD, an outcome presumably reflected in cells/tissues following vaccination.

Key to controlling COVID-19 will be the development of vaccines that provide long-term protection, a property requiring long-term T cell responses [52]. It is known that first- and second-generation adenovirus-based vaccines can elicit strong T cell memory [53] and HD-Ad vaccines share the same capsid proteins. Notably, spleen cell samples from mice 6.5 months after a single intranasal vaccination (5 × 10^9^ viral particles) with HD-Ad_RBD show high levels of antigen-specific IFN-γ (~ 14,000 pg/ml), an indication that it has elicited a strong and long-term T cell response.

The COVID-19 pandemic is being fuelled by emerging variants of SARS-CoV-2 such as the Alpha (B.1.1.7 and Q lineages), Beta (B.1.351 and descendent linages), Gamma (P.1 and descendent linages) and Delta (B.1.617.2 and AY linages). These variants have mutations in the RBD of S protein, which may affect the effectiveness of the currently approved vaccines. In a preliminary study, we measured the serum neutralizing antibody titers of BALB/c mice immunized with HD-Ad_RBD by the prime-boost regimen (Fig. 2A) against three variants that are available to us. We observed ~ 50% reduction in neutralizing antibody titers against Alpha and Beta variants, but no reduction against the Gamma variant (Additional file 1: Fig. S1A). It should be noted that there were large variations among the individual animals (Additional file 1: Fig. S1B) and given the low number of animals used in this experiment, we cannot draw a meaningful conclusion. Nonetheless, this preliminary data appears to be consistent with a recent human study of the Pfizer mRNA vaccine that it was less effective (neutralizing antibody titer reduced by two thirds) at neutralizing the Beta variant [54].

In addition to its superior safety profile, HD-Ad vectors have a large capacity (up to 36 kb) for transgenes, and this makes it possible to deliver large genes or multiple genes in one vector. These features and the demonstrated efficacy of our HD-Ad_RBD vaccine make HD-Ad an ideal platform for the construction of multivalent vaccines targeting SARS-CoV-2 and its emerging variants, work which is now underway in our laboratories.

## Materials and methods

### Virus and animals

The SARS-CoV-2 virus was isolated from local patients in Toronto in March 2020 as described previously [55]. The virus was passaged 4 times and then used for infection experiments. We have confirmed in separate experiments that no mutation occurred in the virus after four passages. The alpha-, beta- and gamma-variant of SARS-CoV-2 were obtained from BEI Resources through an institutional account. All work with infectious SARS-CoV-2 was performed in the Containment Level 3 (CL-3) facilities at the University of Toronto using appropriate protective equipment and procedures approved by the Institutional Biosafety Committee.

BALB/c and K18-hACE2 C57BL/6 mice were purchased from The Jackson Laboratory. The K18-hACE2 mice were bred in house and each mouse was genotyped before use.

### Construction of the HD-Ad_RBD viral vector

A viral vector genome expressing a secreted form of the RBD of the SARS-CoV-2 spike protein was constructed in multiple steps. We started with a plasmid based on pBluscript containing the chicken beta actin gene (CBA) promoter and the poly A signal from the bovine growth hormone gene (BGHpA) and inserted the UbC gene intron 1 into the plasmid between the CBA promoter and the BGHpA by In-fusion cloning. We then inserted the RBD (residues 328 to 528; Additional file 2: Table S1), with the signal peptide of the human cystatin S gene at its 5ʹ end, between the EcoRV and ApaI restriction sites following the UbC intron; this generated a plasmid expressing the secreted form of RBD under the CBA promoter. Finally, we inserted the AscI fragment from this RBD expressing plasmid into the viral vector, pC4HSU-NarD at the AscI site, resulting in a new plasmid, pC4HSU-NarD-RBD for vaccine production.

### Vaccine production

Vaccine production was carried out using 116 cells [56]. A helper-virus, NG163, was used to provide vector DNA replication and the production of viral capsid proteins. The packaging signal sequence in the helper-virus was flanked by two loxP sites. During vector production, the host cells expressed the Cre recombinase which cleaved off the packaging signal of the helper virus. Thus, only HD-Ad vector particles were assembled. The large-scale production of HD-Ad vectors was carried out in suspension cells using 3 l Bioreactors. The vector particles were harvested from the cell lysate and purified through two rounds of CsCl gradient centrifugation.

### Immunization of BALB/c mice

Female BALB/c mice were intranasally immunized with different doses (10^8^ to 10^10^ viral particles) of HD-Ad_RBD or the HD-Ad vector control (HD-C4HSU) in 20 µl of PBS containing 40 µg/ml of DEAE-Dextran and 0.1% LPC. Depending on the immunization regimen, the mice were either euthanized or boosted with the vaccine after 3 weeks. The boosted mice were sacrificed at 3 weeks after the second vaccination. Mouse tissues as well as samples of blood and bronchoalveolar lavage fluids (BALFs) were collected.

### ELISA

Purified RBD protein was used to coat flat-bottom 96-well plates (Thermo Scientific NUNC-MaxiSorp) at a concentration of 1 μg/ml in 50 mM carbonate coating buffer (pH 9.6) at 4 °C overnight. The following day, plates were blocked with solution containing 1% BSA in PBST. Serially diluted mouse sera or BALFs were added and incubated at 37 °C for 1 h. Antibodies including goat anti-mouse IgG horseradish peroxidase (HRP)-conjugated (31430 thermo) and anti-mouse IgA HRP-conjugated (626720 Invitrogen) were diluted 1:5000, or 1:200 in blocking solution. After incubation for 1 h, the plates were washed and developed with 3,3ʹ,5,5ʹ-tetramethylbiphenyldiamine (TMB, 34028, Thermo) for 10 min. The reactions were stopped with 1.0 M H_2_SO_4_ stop solution. The absorbance was measured on a microplate reader at 450 nm (A450). The endpoints of serum or BALF dilutions were calculated with curve fit analysis of optical density values for serially diluted serum or BALF with a cut-off value set to two or three times the background signal.

### Flow cytometry analysis

Left lungs from HD-Ad_RBD vaccinated and control mice were harvested and digested for 45 min at 37 °C in digestion buffer containing liberase 2 μg/ml and Type IV DNase I 25 units/ml. The lung infiltrated cells were cultured with purified RBD protein at 10 μg/ml for 12 h at 37 °C followed by a 6 h treatment with GolgiPlug (BD 555028). After blocking with FcγIII and FcγII receptors antibodies (BD Pharmingen, 553142), cells were stained with live/dead fixable cell stain (Invitrogen 34955), CD44 BV510, CD4 BV711, and CD8a APC-Cy™7 (BD) antibodies. Stained cells were fixed and permeabilized with Cytofix/Cytoperm Fixation/Permeabilization (BD 555028), and then intracellularly stained with anti-IFN-γ APC (BD). Cells were analysed on a Becton Dickinson LSR II CFI (SickKids Flow Cytometry Facility), using Flowjo × 10.0 software.

### SARS-CoV-2 neutralization assay

Heat-inactivated serum was serially diluted (twofold) in DMEM and incubated with 200 TCID_50_ of SARS-CoV-2 for 2 h at 37 °C. For each dilution, there were six technical replicates. After the 2 h incubation, the serum-virus mixture was then incubated with 20,000 Vero-E6 cells supplied with 2% FBS at 37 °C. CPE of each well was examined at day 5. The highest dilution of serum that can protect 50% of cells from SARS-CoV-2 infection is considered as the neutralizing antibody titer, as described in [4].

### SARS-CoV-2 infection of hACE2 mice

Six- to eight-week-old K18-hACE2 C57BL/6 mice (female and male at equal ratio) were intranasally immunized with 5 × 10^9^ viral particles of HD-Ad_RBD or the HD-Ad vector control (HD-C4HSU) in 20 µl of PBS containing 40 µg/ml DEAE-Dextran and 0.1% LPC. The animals were boosted with the same dose of HD-Ad_RBD or HD-Ad, respectively, 3 weeks after the prime vaccination. Three weeks after the boosted vaccination, mice were infected with 1 × 10^5^ TCID_50_ of SARS-CoV-2 in 50 µl of DMEM via the intranasal route. Animal were euthanized at day 1, 3 and 5 post-challenge and samples were collected for further analysis.

### Measurement of viral burden

The infectious virus number was determined by a cytopathogenic efficiency (CPE) assay. Vero-E6 cells (30,000) were seeded into a 96-well plate 1 day before the inoculation. Collected tissues were weighed and homogenized with stainless steel beads (Qiagen, #69989) in 1 ml of DMEM with 2% FBS. Lung homogenates were centrifugated at 3000*g* for 5 min and the supernatants were collected. Serial tenfold dilutions of the lung homogenates were then added to the monolayer Vero-E6 cells. For each dilution, there were six technical replicates. After 5 days of culture, the CPE of each well was examined, and the virus titer (TCID_50_) was calculated according to the Karber method [57] and normalized by the organ weight.

The viral RNA copy number was measured by one-step real-time quantitative PCR (qRT-PCR) as described in [58]. Briefly, collected tissues were weighted and homogenized with stainless steel beads (Qiagen, #69989) in 1 ml of Buffer RLT. The RNA was extracted with the RNeasy Mini kit (Qiagen, #74104). For oropharyngeal samples, swabs were eluted in 500 µl PBS by vortex, and the viral RNA in the PBS eluent was extracted using QlAamp Viral Mini kit (Qiagen, #52906). Primers and TaqMan probes (IDT, #10006890, 10006891 and 10006893) that target the SARS-CoV-2 envelop (*E*) gene were used to detect the genomic/subgenomic viral RNA. The standard curve of Cq-value to viral copy number was generated using serial tenfold dilutions of the *E* gene plasmid DNA template (IDT, #10006896). qRT-PCR was performed with the NEB Luna universal probe kit (E3006) under the following reaction conditions: 55 °C for 10 min, 95 °C for 1 min, and 40 cycles of 95 °C for 10 s and 58 °C for 30 s. The viral RNA copies were determined by converting the Cq-value according to the standard curve.

### Measurement of cytokine and chemokine mRNA levels

RNA of the lung homogenates was extracted and qRT-PCR were performed as mentioned above. Primers used in this experiment are listed in Additional file 2: Table S2. The mRNA level of cytokines and chemokines were normalized to *GAPDH*. Fold change was calculated using the 2^−ΔΔCq^ method by comparing SARS-CoV-2 infected mice to uninfected mice.

### Histological analysis

Hematoxylin and eosin (H&E) staining was performed at the Centre for Phenogenomics in Toronto. Formalin-fixed tissues were embedded into paraffin blocks. Serial sections (5 mm thick) were prepared and they went through the deparaffinization process with three changes of xylene (3 min each) before being rehydrated with four washes of alcohol (100%, 100%, 95%, 70%, 3 min each). Sections were stained with H&E (EMD Chemicals, Canada) and examined using Cytation 5 (BioTek, Canada).

## Supplementary Information

**Additional file 1: Figure S1.** Neutralizing antibody titers against variants of SARS-CoV-2. Sera from the five BALB/c mice prime-boost vaccinated (5 × 10^9^ + 5 × 10^9^) with HD-Ad-RBD (Fig. 2A) were used to measure the neutralizing antibody titers against three variants. SARS-CoV-2 was also included in parallel and compared. (A) The combined data from all five mice. Bars and errors represent the geometric mean with geometric SD. The red dotted lines indicate the limit of detection (LOD) of the assay. (B) Data from individual mice.

**Additional file 2: Table S1**. Codon optimized RBD (residues 328 to 528) of the SARS-CoV-2 Spike Protein. **Table S2.** Primers used in qRT-PCR analysis of cytokine and chemokine mRNAs.

## Data Availability

All data generated or analysed during this study are included in this published article [and its additional information files].

## Abbreviations

SARS-CoV-2: Severe acute respiratory syndrome coronavirus 2
HD-Ad: Helper-dependent adenovirus
S protein: Spike protein
RBD: Receptor binding domain
hACE2: Human angiotensin converting enzyme 2
