# Supplementary figures and images for: Intranasal HD-Ad vaccine protects the upper and lower respiratory tracts of hACE2 mice against SARS-CoV-2
Source: Cell Biosci. 2021 Dec 8;11:202. doi: 10.1186/s13578-021-00723-0 (PMC8653804; doi:10.1186/s13578-021-00723-0)

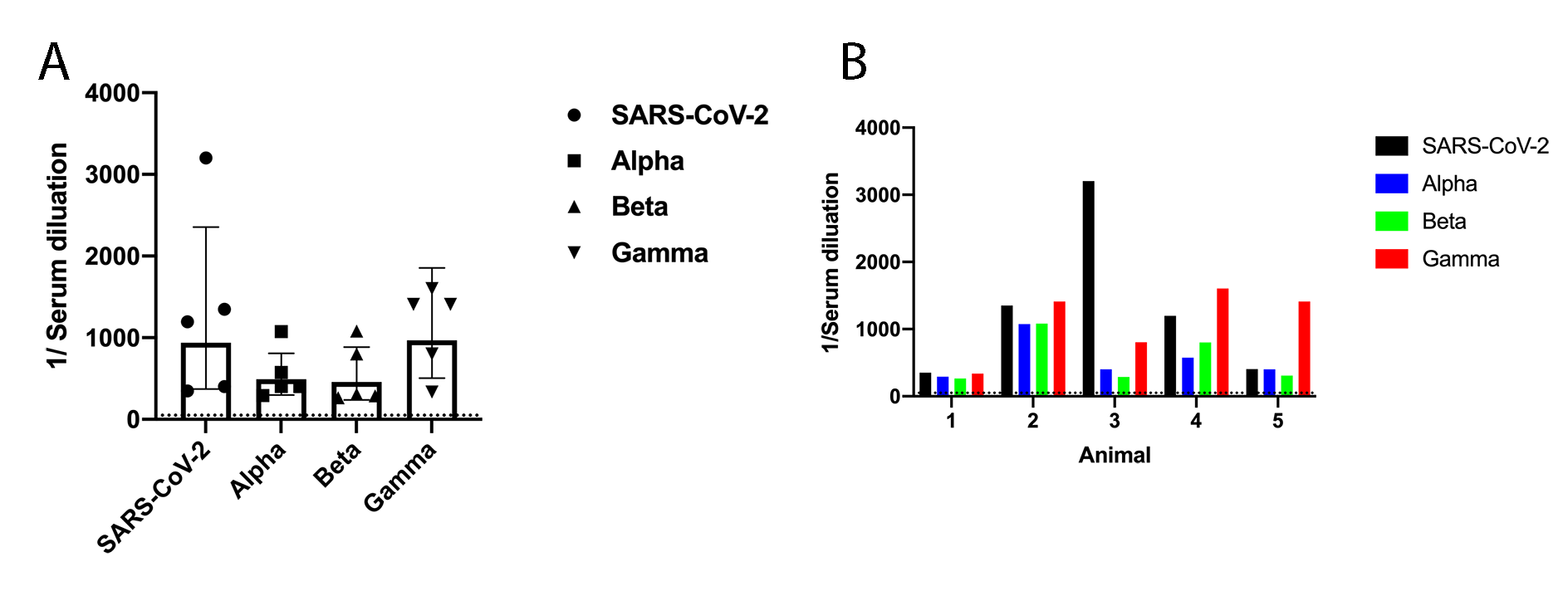

Supplement: Supplementary file 1 — Additional file 1: Figure S1. Neutralizing antibody titers against variants of SARS-CoV-2. Sera from the five BALB/c mice prime-boost vaccinated (5 × 10^9^ + 5 × 10^9^) with HD-Ad-RBD (Fig. 2A) were used to measure the neutralizing antibody titers against three variants. SARS-CoV-2 was also included in parallel and compared. (A) The combined data from all five mice. Bars and errors represent the geometric mean with geometric SD. The red dotted lines indicate the limit of detection (LOD) of the assay. (B) Data from individual mice. [file 13578_2021_723_MOESM1_ESM.tif]
