# Supplementary material for: Intranasal HD-Ad vaccine protects the upper and lower respiratory tracts of hACE2 mice against SARS-CoV-2
Source: Cell Biosci. 2021 Dec 8;11:202. doi: 10.1186/s13578-021-00723-0 (PMC8653804; doi:10.1186/s13578-021-00723-0)
Supplement: Supplementary file 2 — Additional file 2: Table S1. Codon optimized RBD (residues 328 to 528) of the SARS-CoV-2 Spike Protein. Table S2. Primers used in qRT-PCR analysis of cytokine and chemokine mRNAs. [file 13578_2021_723_MOESM2_ESM.docx]

**Table S1. Codon optimized RBD (residues 328 to 528) of the SARS-CoV-2 Spike Protein**

>Cyst1-RBD_328-528 (nucleotide sequence)

ATGGCCCGGCCTCTGTGTACCCTGCTACTCCTGATGGCTACCCTCGCCGGCGCTCTGGCCCGCTTTCCTAATATCACAAACCTGTGCCCATTTGGCGAGGTGTTCAACGCAACCCGCTTCGCCAGCGTGTACGCCTGGAATAGGAAGCGGATCAGCAACTGCGTGGCCGACTATAGCGTGCTGTACAACTCCGCCTCTTTCAGCACCTTTAAGTGCTATGGCGTGTCCCCCACAAAGCTGAATGACCTGTGCTTTACCAACGTCTACGCCGATTCTTTCGTGATCAGGGGCGACGAGGTGCGCCAGATCGCCCCCGGCCAGACAGGCAAGATCGCAGACTACAATTATAAGCTGCCAGACGATTTCACCGGCTGCGTGATCGCCTGGAACAGCAACAATCTGGATTCCAAAGTGGGCGGCAACTACAATTATCTGTACCGGCTGTTTAGAAAGAGCAATCTGAAGCCCTTCGAGAGGGACATCTCTACAGAAATCTACCAGGCCGGCAGCACCCCTTGCAATGGCGTGGAGGGCTTTAACTGTTATTTCCCACTCCAGTCCTACGGCTTCCAGCCCACAAACGGCGTGGGCTATCAGCCTTACCGCGTGGTGGTGCTGAGCTTTGAGCTGCTGCACGCCCCAGCAACAGTGTGCGGCCCCAAG

>Cyst1-RBD_328-528 (protein sequence)

MARPLCTLLLLMATLAGALARFPNITNLCPFGEVFNATRFASVYAWNRKRISNCVADYSVLYNSASFSTFKCYGVSPTKLNDLCFTNVYADSFVIRGDEVRQIAPGQTGKIADYNYKLPDDFTGCVIAWNSNNLDSKVGGNYNYLYRLFRKSNLKPFERDISTEIYQAGSTPCNGVEGFNCYFPLQSYGFQPTNGVGYQPYRVVVLSFELLHAPATVCGPK

**Table S2. Primers used in qRT-PCR analysis of cytokine and chemokine mRNAs**

| *Il11*-RTF (forward) | CTGACGGAGATCACAGTCTGGA |
| --- | --- |
| *Il11*-RTR (reverse) | GGACATCAAGTCTACTCGAAGCC |
| *Cxcl10*_RTF | ATCATCCCTGCGAGCCTATCCT |
| *Cxcl10*_RTR | GACCTTTTTTGGCTAAACGCTTTC |
| *Cxcl11*_RTF | CCGAGTAACGGCTGCGACAAAG |
| *Cxcl11*_RTR | CCTGCATTATGAGGCGAGCTTG |
| *Il-1b*_RTF | TGGACCTTCCAGGATGAGGACA |
| *Il-1b*_RTR | GTTCATCTCGGAGCCTGTAGTG |
| *Il-6*_RTF | TACCACTTCACAAGTCGGAGGC |
| *Il-6*_RTR | CTGCAAGTGCATCATCGTTGTTC |
| *Ifn-γ_*RTF | CAGCAACAGCAAGGCGAAAAAGG |
| *Ifn-γ_*RTR | TTTCCGCTTCCTGAGGCTGGAT |
| *Cxcl1*_RTF | TCCAGAGCTTGAAGGTGTTGCC |
| *Cxcl1*_RTR | AACCAAGGGAGCTTCAGGGTCA |
| *Gadph*_RTF | CATCACTGCCACCCAGAAGACTG |
| *Gadph*_RTR | ATGCCAGTGAGCTTCCCGTTCAG |
